# The Role of Lipids in Retrovirus Replication

**DOI:** 10.3390/v2051146

**Published:** 2010-05-06

**Authors:** Abdul A. Waheed, Eric O. Freed

**Affiliations:** Virus-Cell Interaction Section, HIV Drug Resistance Program, National Cancer Institute, Frederick, MD 21702, USA

**Keywords:** retroviruses, phospholipids, sphingolipids, cholesterol, PI(4,5)P_2_, lipid rafts, entry, assembly, budding

## Abstract

Retroviruses undergo several critical steps to complete a replication cycle. These include the complex processes of virus entry, assembly, and budding that often take place at the plasma membrane of the host cell. Both virus entry and release involve membrane fusion/fission reactions between the viral envelopes and host cell membranes. Accumulating evidence indicates important roles for lipids and lipid microdomains in virus entry and egress. In this review, we outline the current understanding of the role of lipids and membrane microdomains in retroviral replication.

## 1. Introduction

Retroviruses comprise a diverse group of enveloped RNA viruses that cause a variety of diseases, prominent among these are acquired immunodeficiency syndrome (AIDS) and various types of cancers. Retroviruses replicate via the enzyme reverse transcriptase (RT), which produces a linear double-stranded DNA copy of the genomic RNA after entry into the host cell. The newly synthesized viral DNA is then integrated into the genome of the host cell and is replicated as part of the host cell DNA. Retroviruses are classified based on their structure, composition, and replication properties. Retroviral particles are ∼80–120 nm in diameter and their lipid envelope incorporates the viral envelope (Env) glycoproteins that recognize receptors on host cells. Retroviruses encapsidate two identical, linear single-stranded RNA molecules of 7–13 kb in length. Three genes are encoded by the genomes of all replication-competent retroviruses. The *gag* gene encodes the Gag polyprotein precursor, which contains matrix (MA), capsid (CA) and nucleocapsid (NC) domains. For most retroviruses, the *pol* gene encodes the viral protease (PR), RT, and integrase (IN). The *env* gene encodes the surface (SU) and transmembrane (TM) components of the viral Env glycoprotein complex.

Retroviral replication is a multi-step process, with viral entry and budding constituting the two steps that are intimately associated with host cell membranes. Retroviruses enter susceptible target cells by Env-mediated fusion between the viral envelope and the target cell plasma membrane or in low-pH endosomes following endocytic uptake of virions bound to the cell surface. In most cases, virus budding takes place at the plasma membrane of the infected cell and particle release requires a membrane fission, or “pinching-off” reaction whereby the viral and cellular membrane separate. It is well documented that lipids in both the viral and cellular membrane play an important role in retroviral replication; these lipids include cholesterol, sphingolipids and certain phospholipids. In addition to cholesterol and sphingolipids, phosphatidylinositol-4,5-bisphosphate [PI(4,5)P_2_] also serves an important function in viral assembly and release. Studies published over the past decade have provided evidence that cholesterol- and sphingolipid-enriched microdomains in the plasma membrane, known as “lipid rafts”, are involved in both entry and egress of viral particles. In two previous reviews [1,2], we discussed the role of lipid rafts in the replication of both enveloped and non-enveloped viruses. The latter review focused in particular on lipids and membrane microdomains in HIV-1 replication. In this review, we discuss more generally the role of lipids in retroviral replication and provide an update on recent developments in this field.

## 2. Retrovirus classification

Based on their genome organization, retroviruses are informally grouped into two categories: simple and complex. In addition to *gag, pol* and *env* genes that are present in the genomes of simple retroviruses, complex retroviruses encode a diversity of additional proteins. For example, HIV-1 encodes six additional regulatory and accessory proteins, some of which (Vif, Vpr, and Nef) are incorporated in the viral particles and others (Tat, Rev, and Vpu) are not associated with virions (Figure 1). Retroviruses are more formally classified into seven genera based on genome complexity and virion morphology. These genera of the *Retroviridae* include the alpharetroviruses, betaretroviruses, gammaretroviruses, deltaretroviruses, epsilonretroviruses, lentiviruses, and spumaviruses. The first three genera are simple retroviruses, whereas the deltaretroviruses, epsilonretroviruses, lentiviruses, and spumaviruses are complex. The virions of alpha-, gamma-, and deltaretroviruses have a central, spherical core, whereas betaretroviruses have an acentric spherical or cylindrical core. Lentiviruses contain cone-shaped cores while spumaviruses have spherical cores. Some notable examples in each genus include: the alpharetrovirus Rous sarcoma virus (RSV); betaretroviruses mouse mammary tumor virus (MMTV), Jaagsiekte sheep retrovirus (JRSV), and simian type D retrovirus (SRV); the gammaretroviruses murine leukemia virus (MLV), feline leukemia virus (FeLV), and gibbon ape leukemia virus (GALV); the deltaretroviruses bovine leukemia virus (BLV) and human T-lymphotrophic virus (HTLV); and epsilonretroviruses walleye dermal sarcoma virus (WDSV) and snakehead retrovirus (SnRV). The lentivirus genus includes human immunodeficiency viruses (HIV-1 and HIV-2), simian immunodeficiency virus (SIV), feline immunodeficiency virus (FIV), and equine infectious anemia virus (EIAV). Examples of spumaviruses include feline foamy virus (FFV), bovine foamy virus (BFV) and simian foamy virus (SFV).

## 3. Retrovirus replication

Retrovirus replication generally occurs in the following series of steps (Figure 2) [3]: (1) The infection process begins when the SU subunit of the Env glycoprotein complex interacts with its receptor(s) on the target cell. For example, CD4 serves as the major receptor for HIV-1, and the chemokine receptors CCR5 and CXCR4 serve as “co-receptors”. Cell tropism is often determined by the receptor specificity of the Env glycoprotein. In the case of MLV, for instance, ecotropic viruses infect only mouse cells, xenotropic viruses target non-mouse cells, and amphotropic strains infect both mouse and non-mouse cells. (2) Receptor binding induces conformational changes in the Env glycoprotein complex, resulting in exposure of the fusion peptide of the TM protein and its insertion into the target cell membrane. Fusion peptide insertion leads to the fusion of the viral and host cell lipid bilayers. This most often occurs at the plasma membrane but in some cases can take place in a low-pH endosomal compartment. Virion-cell membrane fusion results in the release of the viral core into the cytoplasm of the host cell. (3) In the cytosol of the target cell, the virion core is uncoated and (4) the incoming viral RNA is reverse transcribed by the RT enzyme into a double-stranded DNA copy. (5) The newly synthesized viral DNA, protected within a high-molecular weight “preintegration complex” (PIC), translocates to the nucleus. (6) Once the PIC is inside the nucleus, the viral enzyme IN catalyzes the integration of the viral DNA into the host cell chromosome. (7) The integrated proviral DNA is transcribed into the viral RNAs from the promotor located in the 5′-long terminal repeat (LTR). (8) The viral mRNAs and full-length genomic RNAs are transported from the nucleus to the cytoplasm. (9) The viral RNAs are translated in the cytoplasm and the Gag and GagPol polyprotein precursors traffic to the plasma membrane by poorly understood pathway(s). (10) The Env glycoproteins are translated in the endoplasmic reticulum (ER) and are transported to the plasma membrane via the secretory pathway. (11) Membrane-targeted Gag and GagPol polyproteins recruit the genomic RNAs and assemble at the plasma membrane, leading to the induction of membrane curvature at the site of assembly. For some genera of retroviruses, assembly occurs in the cytosol in the absence of membrane association (e.g., the betaretroviruses) or in the ER (e.g., the spumaviruses). (12) The Env glycoproteins are incorporated into the budding particles during the assembly process. (13) The budding virions wrap themselves in the host cell plasma membrane and pinch off from the surface of the infected cell. Virus budding and release are mediated by the hijacking of cellular endosomal sorting machinery, which normally functions in the sorting of cargo proteins and the budding of vesicles into multivesicular bodies (MVBs). (14) The released virions undergo morphological changes known as maturation, which takes place during or after budding. The maturation process involves viral PR-mediated cleavage of the Gag and GagPol precursors in the virion. With maturation, the viral replication cycle is complete, and the newly released virions can initiate a new round of infection.

## 4. Lipids in cell and viral membranes

### 4.1. Composition of cell membranes

The major structural lipids in mammalian cell membranes are glycerophospholipids (GPLs), sphingolipids, and sterols [2,4–6]. All cellular membranes contain GPLs, and based on the head group attached via glycerol to two acyl chains, GPLs are classified into several types. These include phosphatidylethanolamine (PE), phosphatidylcholine (PC), phosphatidylserine (PS), phosphatidic acid (PA), and phosphatidylinositol (PI). The hydrophobic portion of GPLs is a diacylglycerol (DAG) moiety that contains saturated or *cis*-unsaturated fatty acyl chains of varying lengths (Figure 3). PC is the major lipid in mammalian cell membranes, accounting for ∼50% of the total phospholipid content. Most PC molecules have one *cis*-unsaturated fatty acyl chain. PE, the second most abundant GPL in cell membranes, imposes membrane curvature due to its small polar head group. This curvature helps in budding, fission and fusion of cell membranes [7]. Although GPLs are sufficient to form bilayers, most mammalian cells also contain sphingolipids and sterols. Sphingolipids contain a hydrophobic ceramide (Cer) backbone. The major sphingolipids in mammalian cells are sphingomyelin (SM) and glycosphingolipids (GSLs) that differ in their head group. SM contains a phosphatidylcholine head group, whereas GSLs contain a carbohydrate moiety. Depending on the specific carbohydrate modification, GSLs are further classified into several types, e.g., glucosylceramide (GlcCer) and galactosylceramide (GalCer). Gangliosides are one type of GSLs in which one or more sialic acids are linked to the sugar chain. Sphingolipids in general have saturated (or *trans*-unsaturated) acyl chains, causing them to form long, narrow structures that pack more tightly than GPLs, resulting in a solid gel phase. Sterols are the major non-polar lipids in cell membranes, and cholesterol is the principle sterol in mammalian cell membranes. Sphingolipids and sterols are present at low levels in intracellular membranes compared to the plasma and endosomal membranes [6,8,9]. Sphingolipids are synthesized in the Golgi apparatus, whereas glycerophospholipids and sterols are made in the ER.

The cellular lipidome is made up of a large number of glycerophospholipids, sphingolipids, and sterol-derived molecules [10,11]. Lipid composition varies between cell types, and lipids in the plasma membrane are distinct from those located in intracellular membranes, and among intracellular membranes the composition differs depending on the organelle. The major lipids in mammalian cell membranes are PC, PE, PS, PA, PI, SM, and cholesterol [6,12,13]. Most sphingolipids and PC are distributed predominantly in the outer leaflet of the membrane, whereas PE, PS, and PI are primarily in the inner leaflet [14]. Cholesterol is present in both leaflets and spontaneously flips between the two leaflets [15]. Due to the stronger interactions of cholesterol with sphingolipids and tight packing of highly saturated sphingolipid acyl chains, phase separation occurs in the membranes, resulting in the formation of a liquid-ordered (l_o_) phase [16,17]. Phospholipids are rich in kinked, unsaturated acyl chains and exhibit weaker interactions with cholesterol, resulting in a loosely packed liquid-disordered (l_d_) phase [16,17]. Thus, cholesterol plays an important role in regulating phase behavior, with the l_o_ phase favored at high concentrations [5,17,18].

Due to the enrichment of sphingolipids in the outer leaflet of the plasma membrane, clustering of sphingolipids and cholesterol occurs, resulting in a l_o_ domain that “floats” in the surrounding l_d_ domains. These floating microdomains have been termed lipid rafts (Figure 4) [19–21]. Lipid rafts can be biochemically isolated as detergent-resistant membrane (DRM) at low temperatures due to their insolubility in nonionic detergents like Triton X-100 [22]. DRM complexes float to a low density in sucrose gradients because of their high lipid content [17,18]. Although the DRM approach for isolating lipid rafts has major shortcomings – e.g., DRMs are aggregates of raft domains that are formed after cell lysis [4] – this approach is still commonly used to identify raft-associated proteins [17,18]. To overcome the limitation of DRM-based assays, immunofluorescence microscopy methods that visualize copatching of a protein of interest with well-known raft markers (e.g., GM1) can be coupled with DRM methods to identify protein association with lipid rafts [23,24]. Lipid rafts, or more generally cholesterol-enriched membrane microdomains, have been visualized in living cells by using advanced microscopic approaches including fluorescence resonance energy transfer (FRET), fluorescence recovery after photobleaching (FRAP), single-particle tracking (SPT), two-photon microscopy, and immunoelectron microscopy (IEM) [23,25–32].

### 4.2. Composition of viral membranes

Enveloped viruses acquire their lipid bilayers from host-derived membranes. The origin of the viral membrane depends on the site of virus assembly and budding. For the majority of retroviruses, the plasma membrane is the primary site of membrane envelopment. Interestingly, the lipid composition of viral lipid bilayers is often significantly different from that of the host cell membrane from which the viral envelope is derived. Indeed, it was this observation that first suggested the possibility that enveloped viruses assemble in distinct membrane microdomains [33–35]. For example, the lipidome of retroviral membranes is strikingly different from that of the host cell plasma membrane [36–38]. PC, the major phospholipid in mammalian plasma membranes, is reduced by about two-fold [37,38]. PE in the HIV-1 membrane was shown to be reduced relative to the host cell plasma membrane by two-fold in one study [37] but enriched by 1.6-fold in another report [38]. The lipids that are enriched in viral membranes compared to host cell membranes are SMs (∼2–3 fold), PS (∼2–3 fold), and plasmalogen PE (pl-PE) (∼2 fold) [37,38]. Cholesterol is also enriched in the viral membrane by a factor of ∼2 in virions [36–38]. Cer was observed to be reduced in virions by over three-fold [37,38]. Overall, the HIV membrane is reportedly enriched in saturated PCs by ∼3.6-fold compared with the cell membrane. This enrichment is counter-balanced by a ∼2.5-fold reduction in diunsaturated and polyunsaturated PCs [37]. Interestingly, phosphatidylinositol monophosphate (PIP) and PI(4,5)P_2_ are enriched in the HIV-1 membrane by 1.6-fold compared to the plasma membrane [38], supporting a role for PI(4,5)P_2_ in virus assembly (see below). Significantly, the lipid composition of the HIV-1 membrane is very similar to that of DRMs [37], suggesting that HIV-1 membranes are derived from cholesterol-enriched lipid rafts. The lipidome of the gammaretrovirus MLV was also reported by Wenk and co-workers to contain a higher ratio of cholesterol (two-fold), dhSM (1.8-fold), Cer (2.2-fold), GM3 (2.9-fold), PIP (4.7-fold), and PI(4,5)P_2_ (10.4-fold) compared to the plasma membrane of the producer cells [38]. Again, these studies indicate that the lipid composition of the viral envelope is distinct from that of the plasma membrane, but similar to that of lipid rafts, suggesting that these retroviruses bud from cholesterol-enriched plasma membrane microdomains.

## 5. Lipids in retrovirus replication

### 5.1. Lipids in retrovirus entry

The entry of retroviruses into their target cells is initiated by interactions between viral Env glycoproteins embedded in the lipid bilayer of the virion and specific cell-surface receptors. After attachment to their cellular receptor(s), retroviruses can enter cells by direct fusion at the plasma membrane or by receptor-mediated endocytosis of the virus particle followed by fusion between viral and endosomal membrane. The fusion process is initiated by conformational changes in Env that result in exposure of a hydrophobic fusion peptide at or near the N-terminus of the TM subunit and two heptad repeats downstream of the fusion peptide [3,39]. Fusion of viral and cell membranes is required for the penetration of the viral core into the host cell cytoplasm. Lipids and lipid microdomains on the plasma membrane of the host cell and on the viral membrane play an important role in viral entry. The function of specific lipids in viral entry is discussed in detail below.

#### 5.1.1. Role of cholesterol in retrovirus entry

As discussed earlier, the lipid bilayers of many retroviruses are enriched in cholesterol, which supports their genesis in lipid raft microdomains [36–38]. This cholesterol enrichment appears to be important for viral infectivity. Depleting cholesterol from virions with ß-cyclodextrin (BCD) significantly inhibits the infectivity of HIV-1 virions [40–43]. A limitation of cholesterol depletion approaches is that removal of the majority of cholesterol from HIV-1 virions results in loss of viral components due to the formation of holes in the viral lipid bilayer [41]. However, modest depletion of cholesterol using milder cholesterol sequestering agents such as 2-hydroxypropyl-ß-cyclodextrin (2OHpßCD) does not cause substantial loss of viral proteins, but infectivity is still markedly reduced [40–43]. In this context, replenishment of cholesterol restores the infectivity of HIV-1 indicating that the virions were not completely disrupted after modest removal of cholesterol [40–43]. The effects of cholesterol depletion on virion density were also reversed by cholesterol replenishment [40]. Interestingly, replenishment with the cholesterol analog cholestenone reverses the shift in density induced by cholesterol depletion but does not restore virus infectivity [40]. The infectivity of VSV-G-pseudotyped HIV-1 virions was less susceptible to cholesterol depletion than the infectivity of virions bearing the native HIV-1 Env glycoproteins, suggesting that this sterol is critical for receptor-mediated HIV-1 entry [42]. Furthermore, a number of studies found that HIV-1 particles produced from cholesterol-depleted cells were poorly infectious compared to those produced from normal cells [44–46]. Because virions bud from cholesterol-rich microdomains, cholesterol depletion results in a reduced amount of cholesterol on the viral membrane, which in turn inhibits viral infectivity. Cholesterol-chelating compounds like nystatin have also been reported to inhibit HIV-1 infectivity [42,47]. Recently, we have shown that the cholesterol-binding compound amphotericin B methyl ester (AME), a water-soluble derivative of amphotericin B, inhibits HIV-1 entry [48–51].

Depletion of cholesterol from the target cell inhibits viral fusion and entry, resulting in impaired infectivity [41,52–55]. This inhibition could be due to perturbation of lipid rafts, resulting in dissociation of CD4 or co-receptors from these microdomains. It was reported that both CD4 and CCR5 are associated with lipid rafts [55,56], and that a CD4 mutant that is defective in raft association is unable to support HIV-1 infection in CD4^+^ T-cells [57]. However, studies from other groups suggested that HIV-1 entry is not dependent on the raft localization of CD4 or CCR5 [58,59]. It has been suggested that CXCR4, although normally not raft-associated, is recruited into raft aggregates after gp120 binding [55]. Interestingly, the inhibitory effects of cholesterol depletion can be overcome by coreceptor overexpression [60]. It has also been reported that a highly conserved motif, LWYIK, located near the N-terminus of the membrane-spanning domain of gp41, binds to cholesterol and that mutations in this domain inhibit HIV-1 infectivity [61–63]. In addition to acute cholesterol depletion with cyclodextrins, an alternative approach to limiting the formation of lipid rafts is to inhibit cholesterol biosynthesis using statin drugs, which target the enzyme 3-hydroxy-3-methylglutaryl coenzyme A (HMG-CoA). For example, lovastatin treatment of macrophages significantly inhibits productive HIV-1 entry [64]. Further, drugs like nystatin and filipin that modify the cholesterol organization within cells without affecting overall cholesterol concentration also inhibit HIV-1 entry into macrophages [64]. The impairment of entry is correlated with the reduction in cell surface expression of CD4 and CCR5 [64]. This suggests that, at least in macrophages, cholesterol could potentially enhance the ability of the gp120-gp41 complex to cluster CD4 and coreceptors during the initiation of fusion.

Cholesterol and caveolae – flask-shaped invaginations in the plasma membrane that are categorized as a special type of lipid raft microdomain [65] – have also been implicated in MLV infection. The entry of amphotropic MLV was initially thought to occur directly at the plasma membrane, whereas in most cell types ectotropic MLV was reported to enter by pH-dependent fusion after endosomal uptake [66,67]. However, more recent studies show that amphotrophic MLV also enters target cells through caveolae-mediated endocytosis via its receptor Pit2 [68,69]. Beer *et al*. also found a direct association of Pit2 with caveolin, the protein component of caveolae, as demonstrated by coimmunoprecipitation and inhibition of amphotropic MLV infection by dominant-negative caveolin-1 [68]. The receptor for ectotropic MLV is the cationic amino acid transporter 1 (CAT1), and, based on its colocalization with caveolin, is thought to be associated with caveolae [66,70]. CAT1 is also detected in DRM fractions [71]. It has been reported that depleting cholesterol from target cells that express endogenous or over-expressed CAT-1 resulted in inhibition of MLV entry [71,72]. These studies indicate that cholesterol plays an important role in caveolae-mediated endocytosis of MLV.

There is a considerable amount of evidence to support the hypothesis that HTLV-1 entry is dependent on cholesterol-rich domains. Niyogi and Hildreth reported that monoclonal antibodies that bind to raft-associated proteins, including several GPI-anchored proteins, inhibit HTLV-1-induced syncytium formation [73]. Depletion of cellular cholesterol with BCD inhibited syncytium formation [73] and binding of HTLV-1 virions to T cells [74]. Two of the reported HTLV receptors, GLUT-1 and neuropilin-1, have been shown to colocalize with raft-associated markers and to cluster in specific membrane microdomains [75,76]. Cholesterol-depletion experiments suggest that cholesterol promotes efficient HTLV entry into target cells without affecting receptor binding [76]. These studies support the importance of cholesterol and raft-associated receptors in HTLV-1 entry.

The importance of cholesterol has also been demonstrated for other retroviruses. For EIAV, BCD treatment of the virion but not the target cell interferes with viral infectivity [77]. SIV infectivity is also susceptible to cholesterol depletion [41]. Finally, we have shown that the cholesterol-binding compound AME inhibits the infectivity of SIV by more than 20-fold, similar to its effect on HIV-1 [49–50].

#### 5.1.2. Role of sphingolipids in retrovirus entry

Sphingolipids, like cholesterol, are an important constituent of lipid rafts, and have also been reported to play an important role in viral entry. Several lines of evidence suggest that GSLs in the cell membrane serve to promote HIV-1 binding. It was reported that the V3 loop of gp120 interacts with galactoceramide (GalCer) to facilitate infection of cells lacking CD4 [78–82]. Yahi *et al.* reported that a synthetic peptide derived from the V3 loop consensus motif (GPGRAF) inhibited HIV-1 infection both in CD4-negative and CD4-positive cells [83]. Synthetic soluble analogs of GalCer, and anti-GalCer antibodies, reportedly inhibit HIV-1 entry when present during infection [80,82,84]. Hammache *et al*. demonstrated specific interactions of HIV-1 and HIV-2 Env glycoproteins with GSLs GalCer, GM3, and globotriaosylceramide (Gb3) [81]. A similar study showed preferential interactions of gp120 from CXCR4- and CCR5-tropic viruses with Gb3 and GM3, respectively [85]. Several studies suggest that besides GM3, GM1 also functions as a cofactor for HIV-1 Env-mediated fusion [81,86–89]. Interestingly, reconstitution of GSLs such as GM3 and Gb3 in GSL-depleted GM95 cells restored HIV-1 Env-mediated fusion [88,90]. The galactosyl ceramide/sulfatide was shown to bind directly to gp120 [78,79]. Pretreating CD4^+^ cells with compounds that inhibit GSL synthesis has a significant effect on HIV-1 fusion [88]. The requirement for GSLs in target cells for promoting entry could not be rescued by overexpression of CD4 or coreceptors [87]. Nguyen and Taub showed that co-treatment of target cells with sphingomyelinase (SMase) and cholesterol oxidase reduced HIV-1 infectivity by several fold [91]. SMase treatment of target cells restricted the lateral diffusion of CD4 and inhibited HIV-1 fusion [92]. The upregulation of ceramide, a by-product of SMase treatment, significantly reduced HIV-1 infectivity in CD4^+^ lymphocytes and in monocyte-derived macrophages without overt toxicity [93]. Thus, GSLs may potentially enhance HIV-1 Env-mediated fusion by serving as direct binding sites for Env on the cell surface [89,94]. Furthermore, GSLs may promote the clustering of CD4 and coreceptors in specific domains and regulate their mobility in the target cell membrane, perhaps promoting HIV-1 entry by stabilizing fusion intermediates [89]. In contrast, CD4^+^/coreceptor^+^ derivatives of a mouse melanoma cell line (B16) that expresses exceptionally high levels of GM3 [95] were resistant to CD4-dependent fusion [96]. Interestingly, this block was rescued by pretreating the target cells with GSL inhibitors such as 1-phenyl-2-palmitoylamino-3-morpholino-1-propanol (PPMP); recovery of fusion correlated with downregulation of GM3 to normal levels [96]. The authors proposed that the elevated expression of GM3 in B16 cells could restrict the mobility of CD4 and/or chemokine receptors, thereby blocking the interactions between the different cell surface receptors required for fusion [96]. These observations suggest that GSLs can enhance or inhibit HIV-1 fusion and entry depending on their levels of expression. GSLs could act at different steps in the virus entry process; *i.e.,* they could stabilize virus binding to the cell surface, or could promote the association of CD4/gp120 complexes with co-receptor [97]. Although a role for GSLs in HIV-1 entry is compelling, the mechanism underlying the GSL requirement for viral entry remains to be elucidated.

#### 5.1.3. Role of phospholipids in retrovirus entry

Phosphatidylserine (PS) is one of the membrane phospholipids that is enriched in HIV-1 virions relative to the host cell plasma membrane [36–38]. PS is normally present in the inner leaflet of the lipid bilayer; however, during apoptosis, PS is exposed in the outer leaflet [98]. Following infection with HIV-1, T cells undergo programmed cell death, resulting in translocation of PS to the outer leaflet. Differentiated macrophages, but not monocytes, constitutively express PS in the outer leaflet of their plasma membranes [99]. HIV-1 particles produced from macrophages thus incorporate relatively high levels of PS in the outer leaflet of their envelopes [100]. Recognition of PS in the viral envelope by macrophages is not required for virus binding; however, PS reportedly stabilizes virus-cell interactions, favoring more efficient fusion [100]. Miller and co-workers found that treating cells with PS-containing liposomes increased infection by multiple enveloped viruses by up to 20-fold [101,102]. Treating with PS-enriched liposomes does not alter receptor levels or Env binding to cells, suggesting that PS enhances virus fusion [100]. In some settings, glycosylation of retroviral receptors blocks virus entry, apparently serving as a host-cell defense mechanism against retroviral infection [103]. In this context, treatment of cells with PS liposomes was observed to overcome the infectivity block [101]. Although the mechanism behind this phenomenon awaits clarification, PS treatment does not appear to act by altering the glycosylation state of the receptor. Unlike the rapid enhancement in infectivity conferred by PS treatment in cells containing functional virus receptors, the effect in cells expressing glycosylated receptors occurs with markedly delayed kinetics [101].

### 5.2. Lipids in retrovirus assembly

Retroviral assembly is a multistep process that takes place late in the replication cycle when the viral components come together to form a new generation of virus particles (reviewed in [104–106]). Replication-competent retroviruses typically encode two membrane-associated structural proteins, the Env glycoprotein and the Gag polyprotein precursor. Expression of the Gag precursor alone is sufficient to drive the formation of immature virus-like particles (VLPs), whereas for the production of infectious particles the Env glycoproteins and *pol*-coded enzymes PR, RT and IN are also required. Assembly takes place in a series of discrete steps and are generally promoted by three functional domains within the Gag precursor: the membrane binding (M), Gag-Gag interaction (I), and late (L) domains. As mentioned above, abundant evidence supports the idea that retroviral assembly and release take place in lipid rafts, consistent with the observation that HIV-1 Gag copatches with the raft component GM1 (Figure 5).

#### 5.2.1. Role of cholesterol in retrovirus assembly

The finding that the envelopes of many retroviruses are markedly enriched in cholesterol relative to the host cell plasma membrane suggests that retroviral budding takes place in cholesterol-enriched membrane microdomains [33,36–38,107,108]. The hypothesis that retroviral assembly takes place in lipid rafts is also supported by biochemical analyses showing that the Gag proteins of several retroviruses, including HIV-1 [45,46,109–114], MLV [115], and HTLV-1 [116] are associated with DRMs. Microdomains containing highly multimerized Gag are likely to be denser than classical lipid rafts because of the tight packing of large numbers of Gag molecules [109,111,112]. The exact nature of membrane microdomain(s) with which Gag associates, and how Gag binding alters the properties of such microdomains, remains to be fully understood.

There are several motifs in retroviral Gag proteins required for membrane binding and DRM association. In the case of HIV-1 and many other retroviruses, the association of the Gag precursor with membranes is mediated by a myristic acid moiety covalently linked to the N-terminal Gly of the MA domain, and a positively charged cluster of amino acids located near the N-terminus of the MA domain. Early reports showed that myristylation of Gag is critical for Gag targeting to membrane, as mutating the N-terminal Gly to which the myristate is covalently attached blocks Gag-membrane association [117–119]. It was proposed that the membrane binding of Gag is regulated by a myristyl switch mechanism, whereby the myristate is in equilibrium between a sequestered and an exposed conformation [120,121]. This model is supported by both structural and biochemical evidence [122–126]. Summers and co-workers have demonstrated that the myristyl switch is triggered by multimerization of Gag [126]. Mutating residues near the N-terminus of MA blocks the myristyl switch [122,127]. Pulse-chase analysis suggests that Gag binding to membrane is rapid but DRM association is slower, and Gag association with lipid rafts is enhanced or stabilized by Gag-Gag interactions [45,112]. Although Gag multimerization appears to stabilize Gag-DRM association, studies with an assembly-deficient NC mutant demonstrated that lower-order Gag multimerization is sufficient for Gag binding to membrane and DRM [128].

The functional relevance of the association of Gag with lipid rafts has been demonstrated by studies performed with cholesterol-depleting agents (e.g., BCD), cholesterol biosynthesis inhibitors (e.g., statins), cholesterol sequestering agents (e.g., nystatin), and a cholesterol-binding fungal antibiotic (AME) [45,46,49,129]. Treating virus-producing cells with these cholesterol-disrupting agents impairs HIV-1 particle production [45,46,49,129]. The inhibition of HIV-1 production by cholesterol depletion is due to reduced Gag binding to the plasma membrane and defective higher-order Gag multimerization [114]. Treating Gag-expressing cells with an unsaturated fatty acid that replaces the N-terminal myristate reduces Gag association with DRMs and virus production [130]. Feng *et al*. reported that treating virus-producing cells with α-interferon inhibited HTLV-1 assembly by preventing Gag association with rafts [116]. These studies indicate that lipid rafts play an important role in retroviruses assembly and release.

The HIV-1 [46,113,131] and MLV [46,68,132] Env glycoproteins are reportedly DRM/lipid raft associated. Acylation of proteins, especially involving multiple palmitates, often leads to raft association. Although the Env glycoproteins of HIV-1 [131,133–135], MLV [136,137], and RSV [138] are palmitoylated, there are contradicting reports in the literature regarding the relationship between retroviral Env palmitylation and DRM association. Some studies reported that palmitylation of two Cys residues in the gp41 cytoplasmic tail (CT) is required for Env-DRM association [131,133]; other reports, however, found that palmitylation is not required for Env-DRM association, Env incorporation into virions, or viral infectivity [133,134]. Studies from Clapham’s group showed that Gag-Env interactions are required for Env association with lipid rafts, as mutations in MA or the gp41 CT that block the putative Gag-gp41 interaction prevent Env association with DRM [139]. Furthermore, these authors showed that Gag coexpression is required for DRM association of Env, suggesting that Gag recruits Env into rafts [139]. By using protein colocalization and DRM fractionation assays, Nabel and co-workers observed that HIV-1 Env and the Ebola glycoprotein localize to distinct domains with HIV-1 Gag [140]. Johnson and co-workers using scanning electron microcopy showed that the glycoproteins of HIV-1, VSV, and MLV are recruited to budding sites formed by either HIV-1 or RSV Gag whereas RSV Env is more selective, exhibiting recruitment only to RSV budding sites [141]. In this regard, it has been demonstrated that the CT of gp41 is required for the Env incorporation into virions in a number of T-cell lines and primary cell types [142]. Mutating the palmitylation site in MLV Env diminishes the association of Env with DRM and significantly reduces Env incorporation [132,136]. However, although the palmitylation of Env reduces its expression on the cell surface, palmitylation has no significant impact on syncytium formation or virus replication [132,136].

Nef, an accessory protein encoded by HIV-1, HIV-2 and SIV, plays an important role in the pathogenesis of these primate lentiviruses. The functions of Nef include enhancement of viral infectivity and downregulation of CD4 and MHC class I molecules from the surface of infected cells [143]. Nef undergoes N-terminal myristylation required for membrane binding and DRM association [144,145]. In addition to N-terminal myristylation, basic residues in Nef also contribute to membrane binding and raft association [146]. Nef expression has been reported to increase the levels of cholesterol and GM1 in virions, perhaps contributing to the enhancement of infectivity induced by Nef [145,147]. In macrophages, Nef interacts with ABCA1 (ATP-binding cassette transporter A1) and impairs ABCA1-mediated cholesterol efflux, thereby increasing virion cholesterol content and infectivity [148]. In contrast to this study, Brugger and colleagues observed that Nef increased SM and reduced PC without affecting the levels of cholesterol in virions produced from the MT-4 T-cell line [149]. Nef associates with lipid rafts in which kinases like Lck, Fyn and LAT are enriched, and stimulates p21-activated kinase (PAK) [144,150]. Fackler and co-workers demonstrated that PAK binding and infectivity enhancement require raft association, whereas CD4 downregulation does not; nevertheless, membrane binding is required for Nef function [146,150]. In contrast, Schwartz and co-workers found that not only CD4 downregulation but also viral infectivity is independent of Nef association with lipid rafts [151]. The differences in these studies could be attributed to the use of different cell types or Nef alleles. Additional studies will be required for a more complete understanding of the role of raft association in Nef function.

Vpu is another accessory protein that associates with membranes, though unlike Nef it is not incorporated into viral particles. Vpu significantly enhances HIV-1 release in certain cell types; in these cell types, in the absence of Vpu, virions remain tethered to the plasma membrane after completion of the budding process [152]. Recent studies have identified CD317/BST-2 (also named tetherin) as the host restriction factor that tethers budded virions to the cell surface and is counteracted by Vpu [153,154]. It has been observed that tetherin and Vpu are both raft-associated [155a,155b], further supporting the concept that HIV-1 assembly takes place in lipid rafts.

#### 5.2.2. Role of PI(4,5)P_2_ in retrovirus assembly

PI(4,5)P_2_ plays an important role in the association of HIV-1 Gag with the plasma membrane. PI(4,5)P_2_ belongs to the phosphoinositide family of lipids, the members of which differ in the number and position of phosphates on their inositol head groups [156]. The different phosphoinositide family members are distributed at specific membranes within the cell. For example, PI(3)P is concentrated in early endosomes, PI(3,5)P_2_ is most abundant in late endosomes and PI(4,5)P_2_ is concentrated primarily on the inner leaflet of the plasma membrane [157]. The phosphoinositides help regulate the location of a number of cellular proteins; for example, those that contain pleckstrin homology (PH) and epsin N-terminal homology (ENTH) domains bind directly to the head-group of PI(4,5)P_2_ through their positively charged cluster of basic amino acids [156]. PI(4,5)P_2_ levels can be regulated by either overexpression of a constitutively active ADP-ribosylation factor 6 (Arf6) mutant, Arf6/Q67L, that translocates PI(4,5)P_2_ from the plasma membrane to PI(4,5)P_2_-enriched endosomal structures [158,159] or by phosphatases such as polyphosphoinositide 5-phosphatase IV (5ptaseIV) that hydrolyzes the phosphate at position 5, thereby reducing the levels of PI(4,5)P_2_ in the plasma membrane [160].

It was proposed that the highly basic domain of the HIV-1 MA domain binds to negatively charged lipids in the inner leaflet of the plasma membrane [121,161,162]. Mutating basic residues in the MA domain mistargets Gag, such that it associates with internal compartments rather than the plasma membrane [122,163–169]. These compartments were defined as late endosomes or MVBs [165]. It was shown that overexpression of Arf6/Q67L retargets Gag to PI(4,5)P_2_-enriched endosomal vesicles, whereas overexpression of 5ptaseIV relocalizes Gag from the plasma membrane to late endosmes/MVBs [170,171]. In both cases, due to retargeting of Gag, HIV-1 release is severely reduced. These findings demonstrated that PI(4,5)P_2_ in the plasma membrane is required for efficient HIV-1 release.

Several groups, using different approaches, have demonstrated a direct interaction between MA and PI(4,5)P_2_ [38,124,170,172]. Summers and co-workers used nuclear magnetic resonance (NMR) spectroscopy to solve the structure of a complex between myristylated MA and a soluble derivative of PI(4,5)P_2_ [124,173]. This study suggested that besides electrostatic interactions between the negatively charged headgroup of PI(4,5)P_2_ and basic residues in MA, the 2′-unsaturated acyl chain of PI(4,5)P_2_ binds to a hydrophobic cleft within the globular core of MA (Figure 6) [124,173]. The extrusion of the unsaturated acyl chain from the lipid bilayer upon Gag binding could promote the partitioning of Gag to the more saturated lipid raft microenvironment [124]. Furthermore, these NMR data also suggested that PI(4,5)P_2_ binding to MA triggers the myristyl switch, leading to myristate exposure. Thus, according to this model, PI(4,5)P_2_ acts as both a membrane anchor and a trigger for myristate exposure. Shkriabai *et al.* used mass spectrometric protein footprinting analysis with a lysine-modifying agent to identify MA residues 29 and 31 as being important for the MA-PI(4,5)P_2_ interaction [172]. Notably, mutation of these basic residues mistargets Gag and virus assembly to MVBs [165,167,174,175]. Ono’s group used liposome-binding assays to show that full-length myristylated Gag binds to PI(4,5)P_2_-enriched vesicles, and the residue 29/31 mutant showed reduced PI(4,5)P_2_-dependent liposome binding, again highlighting the importance of these residues in the Gag-PI(4,5)P_2_ interaction [170]. Chan *et al*. reported that HIV-1 and MLV virions are enriched in PI(4,5)P_2_ relative to the plasma membrane, and mutating the basic residues in MA significantly reduced the incorporation of PI(4,5)P_2_ into particles [38]. These observations suggest that MA interacts with PI(4,5)P_2_ not only *in vitro* but also in cells. Direct binding of PI(4,5)P_2_ is also reported for other retroviruses, e.g., HIV-2 [176], EIAV [177], and MLV [178]. Barklis and co-workers recently reported that myristylated MA organizes into hexameric rings of trimers on artificial membranes containing 60% PC, 20% PI(4,5)P_2_, and 20% cholesterol [179]. Interestingly, cholesterol was observed to enhance the selectivity of MA for PI(4,5)P_2_ in this *in vitro* system. Collectively, these studies demonstrate that diverse retroviruses exploit MA-PI(4,5)P_2_ interactions for Gag trafficking and virus release.

### 5.3. Lipids in cell-cell transmission

While most enveloped viruses can infect their target cells as free virions (cell-free infection), in many cases cell-cell transfer is a more efficient mode of virus spread [180–182]. Cell-free infection faces many obstacles, including antibody-mediated neutralization, innate cellular defenses, particle degradation, low target-cell binding efficiency, and the need for virions to diffuse over long distances [182]. Virus transmission directly from infected to uninfected cells can avoid many of these obstacles, thereby increasing the efficiency of virus spread [180,182,183]. A number of studies have demonstrated that retroviruses spread via cell-cell transmission at intercellular contacts known as virological or infectious synapses [174,184–190], which appear to mimic the immunological synapse. The virological synapse, in the case of HIV-1, is formed between the viral Env of the infected cell (effector) and the receptors (CD4, CXCR4/CCR5) on the uninfected (target) cells resulting in lipid raft-like patches on the effector cells (Figure 7) [187,191]. Interestingly, some viruses such as HTLV-1 spread within and between host cells almost entirely by cell-cell transfer because released particles are in general poorly infectious as cell-free virions [192]. Cell-cell transmission is also reported for retroviruses such as HIV-1 [187,189,193] and MLV [194]. Env-receptor interactions appear to be important for synapse formation in some systems, e.g., HIV-1 transfer between T-cells [188] and transfer of MLV across filopodial bridges [194]; however, Env expression is not required for recruitment of Gag to the macrophage-T-cell or macrophage-macrophage synapse [174].

There is accumulating evidence suggesting that the anchoring of filopodia is a prerequisite for the formation of cell-cell interfaces and virological synapses [194–196]. It has been reported that HIV-1 and MLV can drive the formation of filopodia through Env-receptor interactions, then virus particles ‘surf’ on the filopodia towards the uninfected target cell body, where virus-cell fusion occurs in a receptor-dependent manner [194,195]. HIV-1 transmission between T-cells may occur on actin-containing membrane nanotubes that physically connect two or more T-cells over a long distance; the speed with which HIV-1 particles travel between cells is suggestive of actin-mediated transport [196].

It is well established that immunological synapses exhibit raft-like properties [197], and as discussed above, HIV-1 particles enter in and bud from lipid-raft microdomains. It is thus likely that lipid rafts play a role in the formation of virological synapses. Indeed, Sattentau and co-workers found that Env colocalizes with the raft markers GM1 and CD59 at the T-cell synapse in effector cells [191]. Upon conjugation of effector and target cells, within one hour HIV-1 Gag and Env in the effector cell and CD4 in the target cell copolarize to the site of GM1 enrichment. Furthermore, patches of Env and GM1 are also observed on the target cell membrane, suggesting that the fusion of virions with receptors in target cells occur at the virological synapse. Interestingly, upon treating the effector cells with BCD prior to synapse formation, GM1 staining in the effector cells is dispersed, and CD4 polarization in the target cells is prevented [191]. Polarization of Gag and Env to the synapse in the effector cell is also prevented by cholesterol depletion. Derse and co-workers reported that HTLV-1 Gag colocalizes with CD82 and other tetraspanins at the plasma membrane of T-cells, and upon cross-linking with CD82 antibody Gag and CD82 rapidly segregate to patches [198]. Upon cholesterol depletion with BCD, HTLV-1 Gag and CD82 do not segregate efficiently on the cell surface [198]. These studies demonstrate that cholesterol plays an important role in cell-cell virus transmission at the synapse.

## 6. Lipid-modifying agents as inhibitors of retroviral replication

### 6.1. Cholesterol modifying agents

The studies mentioned above reveal the importance of cholesterol in both early and late phases of the virus replication cycle. Modifying the cholesterol content of the virions or target cells inhibits HIV-1 infection [40–42,52,53,55,199,200] and depleting cholesterol from virus-producing cells with BCD or statins inhibits the production of viral particles and the infectivity of the released virions [45,46]. Recent studies from our lab demonstrate that not only cholesterol-depleting agents, but also cholesterol-binding compounds such as AME, have a profound effect on both viral entry and particle production [49,129]. As a result, the replication of HIV-1 and SIVmac239 is inhibited by the presence of AME [49–51]. Truncating the long CT of either HIV-1 or SIVmac gp41 renders the infectivity of virions bearing the truncated Env glycoproteins resistant to AME. Likewise, virions bearing foreign viral glycoproteins from either MLV or VSV are also unaffected by AME in single-cycle infectivity assays. Thus, the presumed binding of AME to cholesterol in the viral envelope blocks particle infectivity in a manner that is specific to virions bearing the full-length HIV-1 Env glycoprotein complex. Incorporation of AME into the viral envelope may perturb membrane fluidity such that the fusogenicity of the full-length Env glycoprotein in suppressed. Interestingly, single-amino-acid substitutions in the HIV-1 gp41 CT confer resistance to this cholesterol-binding compound [49–51]. These mutations create a cleavage site in the gp41 CT for the viral PR, rendering the virus AME-resistant. The inhibitory effect on the release of HIV-1 by AME may be due to impaired ability of the HIV-1 accessory protein Vpu to enhance virus release [129].

The requirement for cholesterol at multiple steps in the HIV-1 replication cycle raises the possibility that cholesterol-depleting or binding agents could potentially offer new approaches to the development of antiviral agents. Statins, which block cholesterol biosynthesis by inhibiting the enzyme HMG-CoA reductase, were examined for antiviral activity *in vivo* in several studies [201–205]. 2-hydroxypropyl-β-cyclodextrin (2OHpßCD) inhibited vaginal transmission of HIV-1 and lovastatin decreased HIV-1 replication in humanized murine model systems [201,202]. Manes and co-workers reported that lovastatin decreased viral loads and increased CD4^+^ cell counts in HIV-1-infected patients [201]. This inhibition is due to down-regulation of Rho GTPase activity by reducing its geranylgeranylation [201]. Inhibition of viral entry and budding in statin-treated cells was reversed by the addition of L-mevalonate or geranylgeranylpyrophosphate, but not by cholesterol. They concluded that the anti-HIV-1 activity conferred by statins is due to inhibition of Rho GTPase activity. In contrast to this report, several other independent studies failed to confirm the antiviral activity of statins *in vivo* [203–205]. Interestingly, Rugeles and co-workers recently showed that in chronically infected individuals lovastatin may display anti-HIV-1 activity [206].

While systemic treatment with drugs that target cholesterol are unlikely to consistently reduce viral loads at subtoxic concentrations, use as chemopreventive agents (“topical microbicides”) may hold more promise. As mentioned above, Markham and co-workers used SCID mice carrying human peripheral blood leukocytes (Hu-PBL-SCID) in which cell-associated HIV-1, but not cell-free virus, was efficiently transmitted by vaginal inoculation of HIV-1 infected cells [202]. They found that topical application of 2OHpßCD to vaginal mucosa prior to inoculation with HIV-1-infected cells blocks transmission of cell-associated HIV-1 without damaging the vaginal epithelial cell membrane.

Lee and co-workers recently reported that an aryl methyldiene rhodanine derivative that intercalates into viral membranes inhibits the infectivity of a wide variety of enveloped viruses. The mechanism of inhibition, however, remains to be defined [207].

### 6.2. Sphingolipid synthesis inhibitors

Several lines of evidence support the concept that sphingolipids play a direct and/or auxiliary role during HIV-1 infection. Although the exact mechanism of action of sphingolipids in HIV-1 infection needs further investigation, these lipids act at the level of virus fusion with their target cells. A number of strategies have been developed to manipulate the levels of sphingolipids or sphingolipid-based molecules to inhibit HIV-1 infection. The levels of sphingolipid can be modulated by inhibiting their biosynthesis at various intermediate steps; for example, l-cycloserine and fumonisin B1 inhibit the activity of serine palmitoyltransferase and dihydroceramide synthase, respectively [89]. L-cycloserine inhibits HIV-1 replication by reducing virion infectivity [208], perhaps in part by down-modulating CD4 expression [209]. The infectivity of virions produced from fumonisin B1-treated MT-4 cells was reduced significantly [37]. Sphingolipids in the viral membrane may play a role in the transfer of HIV-1 mediated by dendritic cells, as virions produced from fumonisin B1-treated cells were inefficiently captured by both immature and mature dendritic cells [210]. PPMP has been shown to block HIV-1 infection in primary T-cells and in various cell lines expressing CD4, CXCR4, and/or CCR5 by blocking Env-mediated plasma membrane fusion and entry [87,90]. While N-butyldeoxynojirimycin inhibits HIV-1 entry, potentially by affecting gp41 exposure [211,212], this compound did not demonstrate efficacy in clinical trials [213]. Metabolites of GSLs such as ceramide, and modified GSLs that preferentially partition into membrane microdomains, were also tested for antiviral activity (reviewed in [89]). Finnegan *et al*. reported that ceramide upregulation in target cells by pharmacological agents such as N-(4-hydroxyphenyl)retinamide (4-HPR, fenretinide), treating cells with SMase, or exogenous addition of long-chain ceramide, can modulate HIV-1 entry [93]. Upregulation of ceramide influences the biophysical properties of the plasma membrane and can initiate various physiological responses such as apoptosis [214]. Kensinger *et al*. synthesized a series of GalCer and SGalCer-derived dendrimers, which mimic the carbohydrate clustering reportedly found in lipid rafts, and one such dentrimer, PS Gal 64mer, inhibited HIV-1 infection [215]. Vierling and coworkers reported that a bipharmacophore that combines the bicyclam AMD3100 and a GalCer analog inhibits HIV-1 replication [216]. Lingwood and colleagues showed that adamantlyGb3, a globotriaosylceramide analog, was able to prevent virus-cell fusion irrespective of HIV strain or chemokine co-receptor preference [217]. These studies suggest that GSL analogs compete with HIV-GSL interactions and inhibit HIV-1 infection [218–220]. If toxicity could be minimized, these compounds might potentially offer novel approaches to antiviral therapy.

## 7. Conclusions and perspectives

It is evident that lipids such as cholesterol and sphingolipids, and membrane microdomains enriched with these lipids, play important roles in both early and late steps in the replication of a large number of enveloped viruses. The involvement of lipid microdomains in retrovirus replication is defined by the association of viral proteins (e.g., Gag, Env, Nef, Vpu), and their cellular partners (e.g., CD4, CCR5, CXCR4, tetherin) with these microdomains. Despite compelling experimental support for the involvement of lipid rafts in retroviral replication, additional information is needed to elucidate the mechanistic basis by which these membrane microdomains promote virus replication. More detailed studies using advanced techniques such as FRET, FRAP, SPT by high-resolution video microscopy, IEM, two-photon microcopy, *etc*., will be useful to obtain further insights. Although the role of lipid microdomains in enveloped virus replication is well established, there are currently no FDA-approved drugs targeting viral lipid envelopes available for the treatment of diseases caused by enveloped viruses. As with all drugs that target host-derived components, adverse side effects of lipid-based drugs are a primary concern. Nevertheless, a more in-depth understanding of the role of lipid microdomains in retroviral replication will not only increase our basic understanding of these important human pathogens, but may also suggest novel approaches for pharmacological intervention.

## Figures and Tables

**Figure 1.**
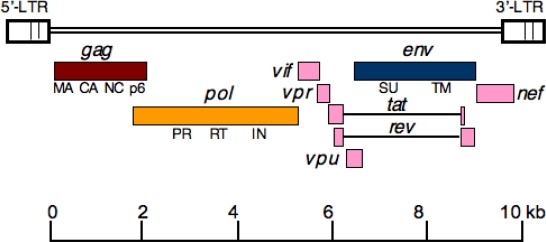
Genomic organization of HIV-1. The rectangles indicate the open reading frames for the *gag* (brown), *pol* (yellow), and *env* (blue) genes. Complex retroviruses like HIV-1 encode additional regulatory and accessory genes (shown in pink). Tat and Rev introns are depicted as horizontal lines. The major Gag domains matrix (MA), capsid (CA), nucleocapsid (NC), and p6 and the Env surface (SU) and transmembrane (TM) glycoproteins (gp120 and gp41, respectively, in the case of HIV-1) are indicated. The 5′ and 3′ long terminal repeats (LTRs) are also shown.

**Figure 2.**
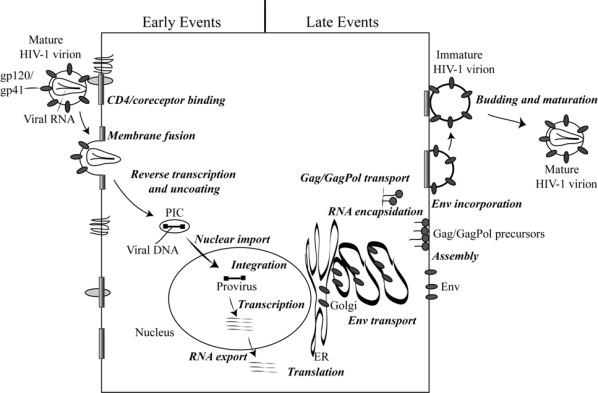
Schematic representation of the HIV-1 replication cycle. The steps that comprise the replication cycle are described in more detail in the text. Reprinted with permission from Freed [221]. Copyright 2004 Elsevier Inc.

**Figure 3.**
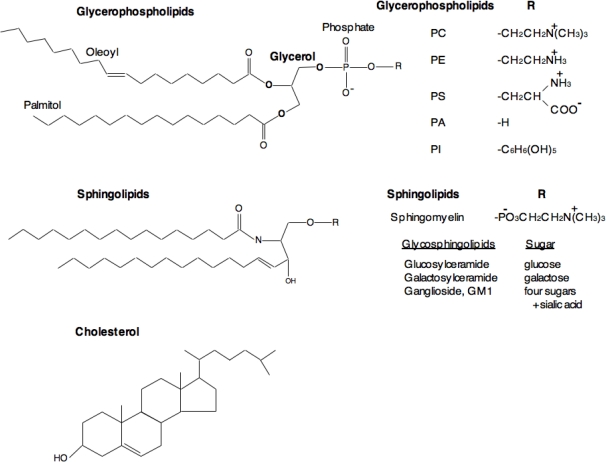
Structures of major classes of lipids in eukaryotic cells. Glycerophospholipids contain diacylglycerol, a phosphate group, and a simple organic head group. The acyl chains contain 16–18 carbon atoms and one of the acyl chains is generally unsaturated with a *cis* double bond. The head groups vary among the phospholipids; e.g., choline in phosphatidylcholine (PC), ethanolamine in phosphatidylethanolamine (PE), serine in phosphatidylserine (PS), and inositol in phosphatidylinositol (PI). Sphingolipids are based on ceramide, which has long saturated acyl chains of 16–26 carbon atoms, and either phosphorylcholine (sphingomyelin) or sugar (glycosphingolipids) headgroups. Depending on the type of sugar, glycosphingolipids are further sub-classified into several types as listed in the figure. Cholesterol is a major sterol in mammalian cells.

**Figure 4.**
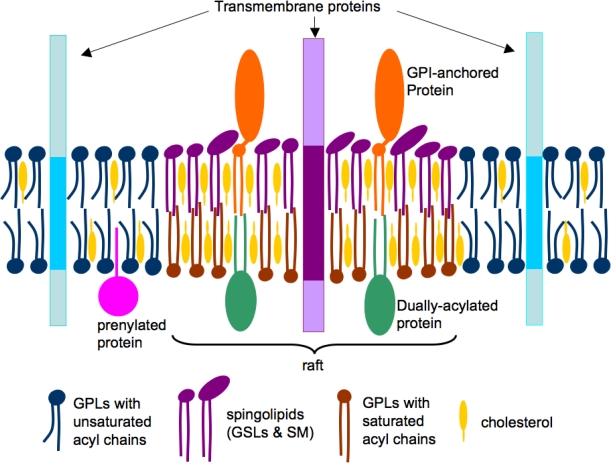
A simplified model of lipid rafts in cell membranes. The phospholipids (blue and brown) and cholesterol (yellow) are distributed in both the leaflets, whereas sphingolipids (violet) are enriched in the outer leaflet of the bilayer. The acyl chains of raft lipids are generally long and saturated (violet and brown), whereas those in non-raft domains are shorter and contain singly or multiply unsaturated acyl chains (blue). Raft domains contain concentrations of dually-acylated (green) and GPI-anchored (brown) proteins, whereas transmembrane (blue) and prenylated (green) proteins are usually non-raft associated. Reprinted with permission from Waheed and Freed [2]. Copyright 2009. Elsevier Inc.

**Figure 5.**
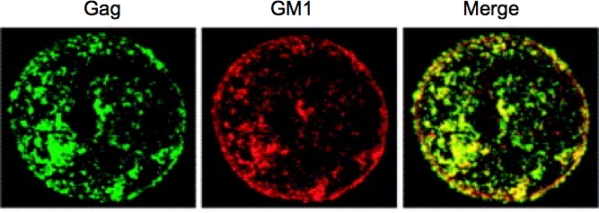
Copatching of HIV-1 Gag proteins with the raft marker GM1. Before fixation, Jurkat cells expressing HIV-1 Gag were treated with cholera toxin B subunit to stain GM1. Cells were then fixed, permeabilized, and Gag proteins were detected with an anti-p17 MA antibody. Gag and GM1 show colocalization, as shown in yellow in the merged image. Reprinted with permission from Ono and Freed [1]. Copyright 2005, Elsevier Inc.

**Figure 6.**
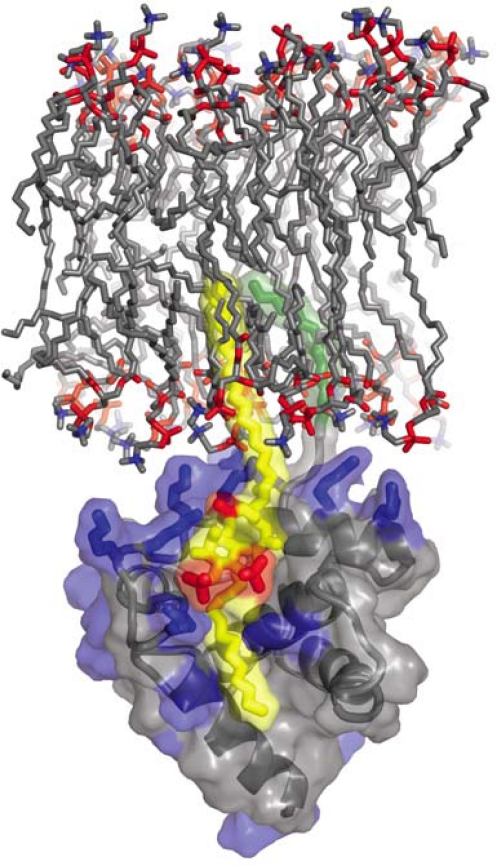
Complex between HIV-1 MA and PI(4,5)P_2_. The highly basic surface of MA (blue) exhibits electrostatic interactions with PI(4,5)P_2_ (yellow and red phosphates). The 2′-unsaturated acyl chain of PI(4,5)P_2_ (yellow) binds to the hydrophobic cleft in MA and the myristyl group (green) of MA inserts into the lipid bilayer. Unpublished image provided by Dr. M. Summers, based on the data of Saad *et al*. [124].

**Figure 7.**
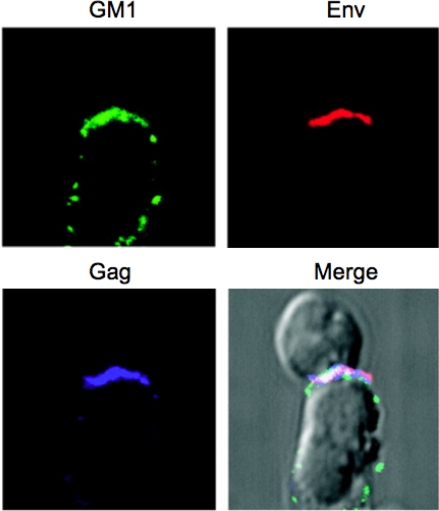
Virological synapse showing HIV-1 Gag and Env concentrating in a raft-rich domain. Primary CD4^+^ T-cells (top) were incubated with HIV-1-infected Jurkat cells (bottom) for 1 h at 37 °C. During the incubation, cells were stained for Env (red), fixed and stained with cholera toxin B subunit (green), specific for the raft marker GM1. Cells were permeabilized and Gag proteins were detected with anti-p17 antibody (blue). Images were superimposed; white indicates the colocalization of three colors. Reprinted with permission from Jolly and Sattentau [191]. Copyright 2005, The American Society of Microbiology.
